# Regulatory Effects of *Lactobacillus crispatus* and *Lactobacillus rhamnosus* on the Formation and Composition of *Gardnerella* Biofilms

**DOI:** 10.3390/microorganisms14030569

**Published:** 2026-03-02

**Authors:** Hanyu Qin, Yun Liu, Sheng Yin, Zhengyuan Zhai, Bingbing Xiao

**Affiliations:** 1 Department of Obstetrics and Gynecology, Peking University First Hospital, Beijing 100034, China; 2 College of Food Science and Nutritional Engineering, China Agricultural University, Beijing 100083, China; 3 Beijing Laboratory for Food Quality and Safety, Beijing Technology and Business University, Beijing 100037, China

**Keywords:** bacterial vaginosis, *Gardnerella* biofilm, *Lactobacillus crispatus*, *Lactobacillus rhamnosus*, probiotics

## Abstract

Bacterial vaginosis (BV), the most common lower genital tract infection among women of childbearing age, is characterized by a decline in *Lactobacillus* populations and the excessive proliferation of anaerobic bacteria. Clinically, metronidazole remains the first-line therapeutic agent. However, the increasing recurrence rate has become an urgent clinical challenge. An important factor of BV recurrence is the persistent presence of *Gardnerella* biofilms, which enhances pathogenic resistance to antibiotics. In contrast, a healthy vaginal microbiome, predominated by *Lactobacillus*, exerts protective effects by producing antimicrobial compounds that inhibit BV pathogen colonization and restore microbial homeostasis. Given this, *Lactobacillus* preparations have gained widespread attention for their adjunctive therapeutic potential in BV management. Accordingly, in this study, we selected two extensively investigated *Lactobacillus* species, *Lactobacillus crispatus* and *Lactobacillus rhamnosus*, to evaluate their inhibitory capacity against *Gardnerella* biofilms. Our findings suggest that hydrogen peroxide and D-lactic acid are prominent bioactive components involved in the inhibition of *Gardnerella* biofilm formation by these *Lactobacillus* species, though the potential contribution of bacteriocins and other uncharacterized factors cannot be excluded. Notably, this inhibitory activity is not accompanied by alterations to the composition of pre-formed biofilms. This study clarifies the anti-biofilm mechanism of specific *Lactobacillus*, providing a valuable reference for future research on probiotic-based strategies for the treatment of BV.

## 1. Introduction

The vaginal microbiome exerts an important influence on reproductive health, with its composition exhibiting dynamic alterations throughout the entire female life cycle [1,2]. The vaginal microbiota of the majority of reproductive-age women can be categorized into five distinct community state types (CSTs). The CST I, II, III, and V are dominated respectively by *Lactobacillus crispatus* (*L. crispatus*), *L. gasseri*, *L. iners*, and *L. jensenii*. In contrast to other CSTs, CST IV exhibits lower *Lactobacillus* abundance and an enrichment of facultative as well as obligate anaerobic microorganisms, such as *Gardnerella*, *Prevotella*, *Fannyhessea* (previously known as *Atopobium*), and *Megasphaera* [3,4]. The vaginal microbiome, dominated by *Lactobacillus*, has been the hallmark of female reproductive health [5]. As the most prevalent microbial taxa in the vaginal microbiome overall, *Lactobacillus* species are capable of synthesizing lactic acid, hydrogen peroxide (H_2_O_2_), and bacteriocins that suppress the colonization of pathogenic microbes and maintain the stability of the vaginal microbiota [6,7]. Lactic acid, including D- and L- isomers, can lower the vaginal pH to 3.5–4.5, resulting in an environment unsuitable for pathogenic microorganisms [8]. However, vaginal *Lactobacillus* species exhibit functional heterogeneity [9]. For example, *L. crispatus* and *L. gasseri* are capable of synthesizing both D- and L-lactic acid, while *L. jensenii* and *L. iners* generate only D-lactic acid and L-lactic acid, respectively [10,11]. These distinct metabolic profiles exert critical impacts on regulating host susceptibility to pathogenic colonization [12,13]. Dysbiosis of the vaginal microbiota is linked to serious obstetric and gynecological complications, including preterm birth [14] and spontaneous miscarriage [15], bacterial vaginosis (BV) [16], vulvovaginal candidiasis [17], as well as an elevated susceptibility to sexually transmitted infections [18,19] (including human papillomavirus, *Neisseria gonorrhoeae*, *Chlamydia trachomatis* and *Mycoplasma genitalium*) and cervical cancer [20].

Bacterial vaginosis (BV) represents the most widespread lower reproductive tract infection in women of childbearing age, with a global prevalence of approximately 30% [21,22]. Notably, it is a definitive example of vaginal microbiome dysregulation driving disease pathogenesis [23]. The vaginal microbiota of BV is distinguished by a marked decrease in vaginal *Lactobacillus* populations alongside the overabundant growth of anaerobic bacteria, similar to CST IV [24,25]. Metronidazole serves as the standard first-line therapeutic agent for BV [26]. Although clinical resolution can be achieved in certain cases, more than 50% of treated patients suffer from BV recurrence within a one-year period [27,28,29]. A potential explanation for BV recurrence involves the formation of *Gardnerella* biofilms [30,31]. On the one hand, the biofilms act as a protective sanctuary for pathogenic bacteria, substantially impairing antibiotic penetration and thus hindering the complete eradication of the infection [21,32,33]. On the other hand, metronidazole therapy drives vaginal microbiota composition toward dominance by *L. iners*, instead of *L. crispatus* [34,35]. Given that *L. iners* produces only L-lactic acid, the vaginal microbiota dominated by it may be insufficient to maintain stability and promote recurrence [36]. Several studies have demonstrated that adjunctive non-antibiotic interventions—including vaginal microbiota transplantation and live biotherapeutic products containing *L. crispatus*, represent promising alternatives to metronidazole monotherapy; even so, BV recurrence remains a prevalent issue [28,37].

Currently, there are no therapies promoting *L. crispatus* dominance in the vaginal microbiota [38]. Therefore, adjuvant therapy involving optimal *Lactobacillus* strains or the targeted elimination of *Gardnerella* biofilms could help lower BV recurrence rates and improve clinical cure rates. As common probiotic strains, *L. crispatus* and *L. rhamnosus* are extensively utilized in the treatment of reproductive and urinary tract infections [39]. Thus, the primary objective of this study was to systematically investigate the inhibitory effects exerted by *L. crispatus* and *L. rhamnosus* on *Gardnerella* biofilms, with a particular emphasis on alterations in *Lactobacillus* metabolic products and the compositional characteristics of *Gardnerella* biofilms. The objective of this research was to provide experimental evidence to support the development of therapeutic strategies centered on microbes that can efficiently reestablish a balanced vaginal microbiota and improve the long-term prognosis of patients with BV.

## 2. Materials and Methods

### 2.1. Study Population and Ethical Approval

Premenopausal women (aged 18–50 years) were recruited in the Department of Gynecology at Peking University First Hospital (Beijing, China) and provided written informed consent. The participants included patients with BV and healthy women. BV can be diagnosed based on Amsel’s criteria [40], or Nugent score [41]. Eligible BV patients were required to meet at least three of the four Amsel diagnostic criteria (homogeneous thin discharge, clue cell positivity, vaginal fluid pH exceeding 4.5, and a positive whiff test) or possess a Gram stain-based Nugent score of 7–10 [26]. The healthy participants had a Nugent score of 0–3 and no symptoms, such as vulvovaginal itching, burning, irritation, odor, or discharge. Participants who met any of the following criteria were excluded from the study: menstruation, pregnancy, or other reproductive tract infectious diseases, such as vulvovaginal candidiasis, aerobic vaginitis, *trichomonas* vaginalis, *Chlamydia trachomatis*, *Mycoplasma*, human papillomavirus infection, cervicitis, and pelvic inflammatory disease. The protocol of this study was reviewed and approved by the Ethics Committee of Peking University First Hospital (approval code: V2.0/2020.04.20), and the research was implemented in adherence to the tenets of the Declaration of Helsinki.

### 2.2. Strain Isolation, Identification, and Storage

Vaginal samples were obtained from the lateral vaginal wall using two swabs. One vaginal specimen was dispatched to the microbiology lab to undergo Gram staining procedures. For the other sample, it was inoculated on sheep blood agar plates to isolate *Gardnerella* and *Lactobacillus* strains, and subsequently incubated at 37 °C in an anaerobic environment for a duration of 48 to 72 h. Suspicious colonies were subjected to further purification and cultivation for 48–72 h. Next, we performed Gram staining and observation under an optical microscope. The *Gardnerella* strain exhibited a homogeneous morphology of short rod-shaped bacteria, while the *Lactobacillus* strain displayed a uniform morphology of Gram-positive, large rod-shaped bacteria.

After several rounds of purification culture, single colonies were harvested from plates and resuspended in 20 μL sterile ddH_2_O. The suspension was mixed thoroughly with a pipette, microwaved at high temperature for 3 min, and chilled in an ice bath for 2 min. The resulting lysed bacterial suspension was used as the colony PCR template. Isolate species identification was performed via 16S ribosomal DNA (rDNA) amplification with universal primers: 16S rDNA-Forward (5′-AGAGTTTGATCCTGGCTCAG-3′) and 16S rDNA-Reverse (5′-TACGGTTACCTTGTTACGACTT-3′). PCR conditions included 30 cycles of pre-denaturation (98 °C, 3 min), denaturation (98 °C, 30 s), annealing (52.8 °C, 30 s), and extension (72 °C, 50 s), plus a final extension at 72 °C for 10 min. The PCR amplification was performed in a 25 μL reaction system, and the PCR amplification product volume was 25 μL. A 5 μL aliquot of the product was subjected to 1% agarose gel electrophoresis to verify the target fragment size [42]. Sequencing of the qualified amplicons was performed by Beijing Qingke Xinye Biotechnology Co., Ltd. (Beijing, China). The resultant sequencing data were submitted to the online NCBI GenBank database, and BLAST (https://blast.ncbi.nlm.nih.gov/Blast.cgi, accessed on 20 August 2021) alignment was conducted to confirm bacterial species. Notably, 16S rDNA sequencing failed to achieve species-level identification for the clinically isolated *Gardnerella* strains, which were therefore collectively categorized as *Gardnerella* species. *Lactobacillus* species were also identified by the same method.

Subsequent to purification and species-level identification, all strains were preserved at −80 °C in microbial storage tubes. Prior to experimentation, each individual strain was revived and re-purified to confirm its viability and purity. A total of 24 *Gardnerella* strains (named *Gardnerella* 1 through 24) and 30 *Lactobacillus* strains (named *Lactobacillus* 1 through 30) were obtained for further analysis.

### 2.3. Determination of Gardnerella Biofilm Formation

The single colonies of *Gardnerella* on the plate were inoculated into supplemented brain–heart infusion broth (sBHI) medium and incubated at 37 °C under anaerobic conditions for 48 h. The bacterial cultures were then normalized to a turbidity of 0.5 McFarland units with sterile phosphate-buffered saline (PBS), and the turbidity was verified using a turbidimeter with reference to a standard McFarland calibration curve. The bacterial suspension (2 µL) was inoculated into 198 µL of supplemented Brain-Heart Infusion (sBHI) broth (composed of 9.25% BHI, 0.3% glucose, and 0.3% soluble starch) and incubated anaerobically at 37 °C in 96-well polystyrene microplates (Corning, New York, NY, USA) for 48 h. All plates were tissue culture (TC)-treated to ensure optimal cell adhesion and bacterial biofilm formation. After cultivation, the bacterial solution was discarded, and the planktonic bacteria were removed by gently rinsing thrice with PBS. Biofilms adhered to and persisted on the surface of the wells. The wells were subjected to air-drying for 60 min prior to staining. A volume of 200 µL of 0.4% crystal violet was added to each well and incubated for 30 min to stain the biofilms. Unbound crystal violet was washed away with 200 mL of PBS via gentle rinsing, and the wells were air-dried for 5 min. The retained crystal violet was then dissolved using 200 mL of 33% acetic acid per well. The absorbance of stained biofilms was measured at 595 nm wavelength using a microplate reader (Infinite M200 PRO, TECAN, Männedorf, Switzerland). The sBHI medium without bacteria was used as control. For each bacterial strain, three independent biofilm formation experiments were conducted, with the average of the triplicate measurements documented.

### 2.4. Scanning Electron Microscopic Observation of Gardnerella Biofilm

The present study aimed to observe the microstructural characteristics of *Gardnerella* biofilms. Therefore, we selected four strains of *Gardnerella* for examination (G1 and G2 with strong ability to form biofilms, as well as G23 and G24 with weak ability to form biofilms). The turbidity of the bacterial suspension was adjusted to 0.5. Samples were cultured in 24-well plates, each containing a 14 × 14 mm circular coverslip at the bottom, under anaerobic conditions at 37 °C for 48 h. Planktonic bacteria in each well were then gently removed, leaving the biofilm adhered to the surface of the coverslip. Next, the cover glasses were washed thrice with sBHI to remove planktonic bacteria and loose impurities. Samples were fixed in 3% glutaraldehyde at 4 °C overnight, subjected to gradient dehydration with an ethanol series, and treated with t-butyl alcohol for substitution prior to freeze-drying. The dried specimens were sputter-coated with platinum using an automatic fine coater and subsequently examined under a scanning electron microscope (SEM) (HITACHI S-3400, JEOL, Tokyo, Japan).

### 2.5. Determination of Biofilm Formation in Co-Cultures of Gardnerella and Lactobacillus

Based on the determination results of *Gardnerella* biofilms, the *Gardnerella* 1 (G1) strain with the strongest biofilm formation ability was selected to investigate the inhibitory effect of *Lactobacillus* on *Gardnerella* biofilms. The G1 strain was separately co-cultured with 30 strains of *Lactobacillus*. The turbidity of the isolated strains was adjusted to 0.5 using a turbidimeter (Xingbai Biotechnology Co., Ltd., Shanghai, China). A mixture containing 2 µL *Gardnerella* suspension, 2 µL *Lactobacillus* suspension, and 196 µL sBHI broth was prepared and transferred to 96-well plates, then incubated under anaerobic conditions at 37 °C for 48 h. The staining and determination for biofilm were performed as previously described (Section 2.3). The biofilm formed by the G1 strain without *Lactobacillus* was used as control. The biofilm biomass was quantified by measuring OD_595_ absorbance. Each strain was subjected to triplicate trials, and the resultant mean value was used for subsequent analysis. To improve data interpretability, the results were normalized to the control group (set as 100% biofilm formation). The biofilm suppression rate was calculated as follows: (1 − OD_595_, treatment/OD_595_, control) × 100%.

### 2.6. Biofilm Formation in Co-Cultures of Gardnerella and Lactobacillus by Confocal Laser Scanning Microscopy

We also selected four *Lactobacillus* strains, *L. rhamnosus* (L1) and *L. crispatus* (L2) (with strong biofilm-inhibiting activity) as well as *L. rhamnosus* (L28) and *L. crispatus* (L29) (with weak biofilm-inhibiting activity) to examine the destructive effect of *Lactobacillus* on *Gardnerella* biofilm. These four *Lactobacillus* strains were respectively co-cultured with the G1 strain at a 1:1 inoculum ratio in 2 mL of sBHI medium. The turbidity of *Lactobacillus* and *Gardnerella* suspensions was adjusted to 0.5. And the biofilm of G1 strain was as control group. Circular cover glass (14 × 14 mm) was placed at the bottom of the well plate. Subsequently, *Lactobacillus* and *Gardnerella* were co-inoculated into 24-well plates and incubated anaerobically at 37 °C for 48 h. The staining solution was prepared with 3 µL of propidium iodide, 3 µL of SYTO^®^9, and 1 mL of sBHI. The cover glass was washed thrice with sBHI and incubated for 25 min with 300 μL of fluorescent stain in the dark at 25 °C. Next, it was gently washed thrice with sBHI to remove the excess stain. The cover glass was placed upside down in a 20 × 20 mm dish. The Petri dish was filled with approximately 1000 µL of sBHI broth, such that the liquid covered the glass by a 2 mm depth, prior to observation under confocal laser scanning microscopy (CLSM) (LSM510, Zeiss, White Plains, NY, USA). We observed the biofilm under 63× magnification with a 0.75 zoom oil objective lens, using a spectral laser (green, 488 nm; red, 561 nm) for excitation light. Axial scanning was performed along the *z*-axis at an interval of 1 μm to acquire a stack of images corresponding to each layer of the sample. The distribution and ration of live and dead bacteria in the biofilm was analyzed using the ImageJ2 software (Version: 2.16.0/1.54p).

### 2.7. Determination of Biofilm Components from Lactobacillus and Gardnerella Co-Culture

The extracellular polymer substances (EPS) from the biofilm formed by co-culture of G1 strain and *Lactobacillus* strains (i.e., L1, L2, L28, and L29) were extracted and their components were analyzed. Initially, the turbidity of each bacterial suspension was adjusted to 0.5. Standardized bacterial suspensions were inoculated into 6-well plates and incubated anaerobically at 37 °C for 48 h to facilitate biofilm formation. Next, the bacterial suspension was removed, and each well was gently rinsed thrice with sBHI to clear non-adherent cells. The plate was then inverted on sterile filter paper for 30s to remove residual moisture. A sterile scraper was used to collect the biofilm, which was weighed immediately to determine the wet biofilm biomass. The collected biomass was then transferred to a centrifuge tube containing sterile PBS. The tube was vortexed for 1–2 min. Finally, the suspension was centrifuged at 3000 rpm for 10 min at 4 °C. The culture supernatant was collected and filtered through a 0.22 μm membrane filter to obtain the biofilm EPS samples. The concentration of polysaccharides, proteins, and deoxyribonucleic acid (DNA) in each sample was calculated. The concentration is expressed as mg per gram of wet biofilm biomass (mg/g). Three experiments were performed for each strain and the average value of the three experiments was recorded. Of note, the methods used quantify total protein/polysaccharides/DNA in the biofilm, without distinguishing contributions from *Gardnerella* and *Lactobacillus*.

#### 2.7.1. Polysaccharide

Determination of polysaccharide content was performed according to the phenol-sulfuric acid method. An aliquot was used to appropriately dilute the EPS solution into a test tube. Subsequently, 6% phenol, 98% concentrated sulfuric acid, and distilled water were added sequentially in a 1:1:5:1 ratio. Next, the mixture was subjected to vigorous shaking for rapid homogenization, and the tube was incubated in a boiling water bath for 15 min to allow the color reaction to proceed to completion. After heating, the mixture was cooled to room temperature naturally and allowed to stand for 5–10 min to stabilize the reaction system. The absorbance of each sample was measured at 495 nm wavelength, and polysaccharide concentrations were calculated based on the glucose standard curve. The dilution factor was adjusted to determine the total polysaccharide content in the original EPS.

#### 2.7.2. Protein

The protein concentration was quantified using the Bicinchoninic Acid (BCA) Assay (Beyotime Biotechnology, Shanghai, China) according to instructions. Initially, BCA working reagent were prepared and stored in the dark for immediate use. Bovine Serum Albumin (BSA) was selected as the external standard, and a series of dilutions were prepared using the identical lysis buffer. Briefly, 20 μL aliquots of each standard solution and protein sample were loaded into a 96-well microplate, with all determinations performed in triplicate. Subsequently, each well was supplemented with 200 μL of the working reagent, and the mixture was homogenized fully using a plate shaker for a 30 s duration. Following mixing, the plate was incubated at 37 °C for 30 min in the dark to allow for color development. After the plate was cooled to room temperature, absorbance values were measured at a wavelength of 562 nm using a microplate reader (Infinite M200 PRO, TECAN). The protein concentration of the unknown samples was calculated based on the linear regression equation derived from the BSA standard curve. All samples were diluted where necessary to ensure the absorbance fell within the linear range of the assay.

#### 2.7.3. DNA

Determination of double-stranded DNA (dsDNA) concentration was performed using the Qubit™ dsDNA HS (High Sensitivity) Assay Kit (Tiangen Biochemical Technology Co., Ltd., Beijing, China) according to the protocol. Qubit working solution was prepared by diluting the Qubit dsDNA HS reagent at a ratio of 1:200 in the supplied buffer. For the calibration step, two standard solutions were prepared by mixing 10 μL of each standard with 190 μL of the prepared working solution. For each unknown sample, 1–10 μL aliquots of DNA were mixed with the working solution, with the final volume of each reaction system set to 200 μL. All assay tubes were vortexed for 2–3 s and subsequently was incubated at room temperature in the dark for 2 min. Fluorescence was measured using a Qubit Fluorometer (Thermo Fisher Scientific, Waltham, MA, USA), and final concentrations were calculated based on the calibration curve generated from the standards.

### 2.8. Compositions of Lactobacillus Supernatant

The *Lactobacillus* strains (L1, L2, L28 and L29) were cultivated in the de Man, Rogosa and Sharpe (MRS) liquid medium at 37 °C for 48 h. After cultivation of the *Lactobacillus* strains, the fermentation broth was subjected to centrifugation (8000 rpm, 10 min, 4 °C) and subsequently filtered through a 0.22 µm membrane to obtain the cell-free supernatant. Triplicate experiments were carried out, and the mean of these three replicates was recorded as the final data point.

#### 2.8.1. pH Value

The pH value of the *Lactobacillus* supernatant was determined using a calibrated digital pH meter. To ensure the reliability and reproducibility of the data, all measurements were performed in biological triplicate. The pH meter was rinsed with deionized water and blotted dry between successive readings to prevent cross-contamination.

#### 2.8.2. H_2_O_2_

Quantification of H_2_O_2_ levels in the *Lactobacillus* supernatant was performed using a Hydrogen Peroxide Assay Kit (Beyotime Biotechnology, Shanghai, China) following the instructions. In a 96-well plate, 50 μL of the supernatant or standard solution was combined with 100 μL of H_2_O_2_ detection reagent. The resulting mixture was subjected to gentle agitation and incubated in the dark at room temperature for 30 min, followed by absorbance determination at 560 nm with a microplate reader (Infinite M200 PRO, TECAN). H_2_O_2_ concentrations were calculated based on the standard curve constructed using H_2_O_2_ solutions of known concentrations and all measurements were performed in triplicate.

#### 2.8.3. D- and L-Lactic Acid

The levels of D-lactic acid and L-lactic acid in the *Lactobacillus* supernatant were quantified enzymatically with the D-/L-Lactic Acid Assay Kit (K-DLATE, Megazyme, Bray, Ireland), following the instructions. The assay was performed in cuvettes containing buffer, NAD^+,^ and D-glutamate-pyruvate transaminase (D-GPT). The initial absorbance (A_1_) was measured at 340 nm. D-lactic acid was quantified by measuring the absorbance change (A_2_) after the addition of D-lactate dehydrogenase (D-LDH). Subsequently, L-lactic acid was determined by measuring the further absorbance increase (A_3_) following the addition of L-lactate dehydrogenase (L-LDH). The absorbance differences (ΔA) for both D- and L-isomers were corrected against a reagent blank, and the final concentrations were calculated based on the molar extinction coefficient of NADH. All measurements were performed in triplicate.

### 2.9. Inhibitory Effect of Lactobacillus Supernatant on Gardnerella Biofilm

Similarly, the supernatants of *Lactobacillus* (L1, L2, L28, and L29) strains were extracted. Next, the supernatants were added at different stages of G1 biofilm formation. The G1 bacterial suspension was adjusted to a turbidity of 0.5. Next, 1 µL of G1 bacterial suspension and 100 µL of sBHI medium were inoculated into a 96-well plate, and cultured anaerobically at 37 °C. The *Lactobacillus* supernatant (100 μL) was added separately at 0, 24, and 48 h of G1 medium, followed by continued incubation for 48 h. For the control, the supernatant was replaced with 100 μL of sterile PBS. PBS served as a neutral control to avoid introducing extra nutrients, ensuring that the inhibitory effects resulted solely from the *Lactobacillus* supernatants. As described previously, the absorbance value of the biofilm was quantified at a wavelength of 595 nm (Section 2.3). Three experiments were performed for each strain and the average value of the three experiments was recorded.

### 2.10. Statistical Analysis

Statistical analyses were performed using SPSS software (Version 19.0, IBM Corp., Armonk, NY, USA). Analysis of variance (ANOVA) was performed to analyze the quantification of biofilm, differences in *Gardnerella* biofilm composition and *Lactobacillus* supernatants among different groups. And ANOVA was also used to analyze the difference in *Gardnerella* biofilm quantification at different *Gardnerella* culture time stages with addition of *Lactobacillus* supernatants. Statistical significance was defined as a *p*-value of 0.05 or less.

## 3. Results

### 3.1. Biofilm Formation of Gardnerella Strains

A total of 24 *Gardnerella* strains were isolated from vaginal secretions of patients with BV. While 16S rDNA sequencing enables preliminary identification of *Gardnerella*, it is insufficient to distinguish between different species within the *Gardnerella* genus or genotypes within a single species. For this reason, all isolated strains were collectively designated as “*Gardnerella* strains” in or study.

**Figure 1:**
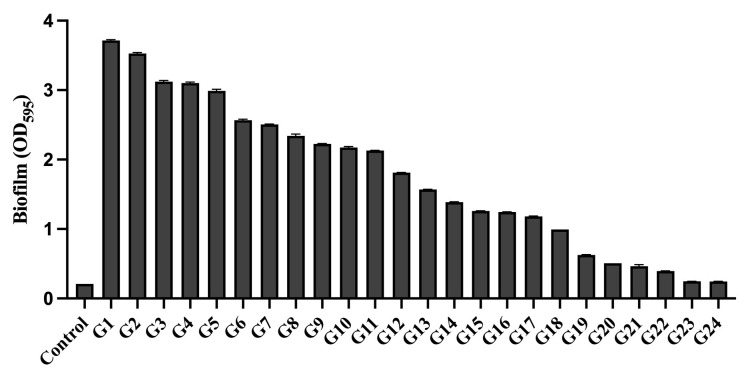
Quantitative analysis of biofilm formation by 24 *Gardnerella* strains. The images are arranged in descending order of biofilm quantity from high to low, with strains numbered corresponding to this ranking. One-way ANOVA indicated significant differences in biofilm biomass among *Gardnerella* strains (*p* < 0.05). Dunnett’s multiple comparisons test was performed using the control group as the reference to identify significant differences in biofilm biomass between each *Gardnerella* strain and the control (*p* < 0.05).

We separately quantified the biofilm-forming capacity of these 24 strains. The strains were numbered and labeled according to the biofilm formation quantity, sorted in descending order of their biofilm quantitative values (Figure 1). The results showed statistically significant differences in biofilm formation among the 24 strains of *Gardnerella* (*p* < 0.05), suggesting heterogeneity in biofilm formation ability. The raw data are available in the supplementary materials.

### 3.2. Scanning Electron Microscopy Observation of Gardnerella Biofilm Structure

**Figure 2:**
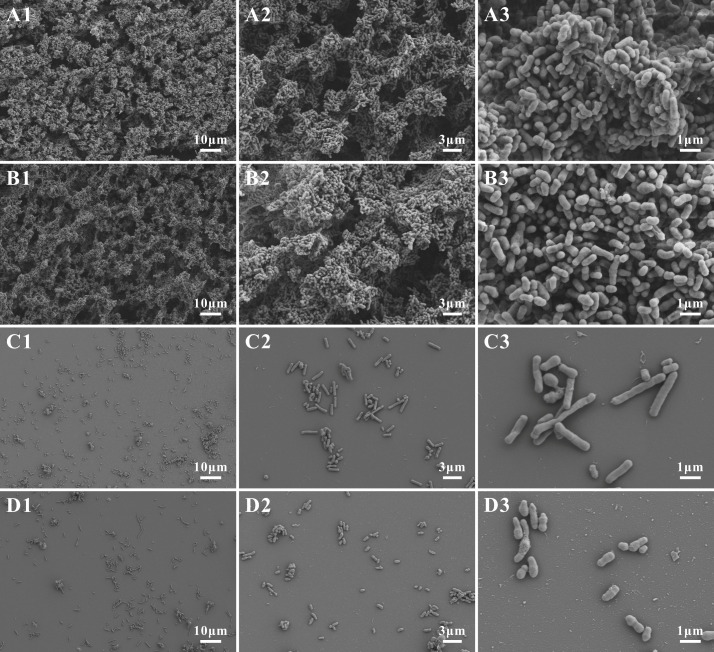
Scanning electron microscopy observation of biofilm formation by *Gardnerella*. (**A1**–**D3**) correspond to strains G1, G2, G23, and G24, respectively. Magnifications are indicated as follows: 1 = 1.00 K×, 2 = 3.00 K×, 3 = 10.00 K×.

We selected four *Gardnerella* strains, namely G1 and G2 (with strong biofilm-forming ability) and G23 and G24 (with weak biofilm-forming ability), for comparative ultrastructural analysis using SEM. The results are presented in Figure 2. G1 and G2 formed confluent biofilm aggregates, where bacterial cells were extensively interconnected (Figure 2A,B). In contrast, G23 and G24 biofilms displayed sparse colonization patterns, characterized by isolated single bacterium and small microcolonies (Figure 2C,D). These observations confirm significant inter-strain heterogeneity in the structural characteristics of *Gardnerella* biofilms.

### 3.3. Lactobacillus Strains

We isolated a total of 30 distinct *Lactobacillus* strains from vaginal specimens of healthy female subjects. Species identification revealed the following distribution: *L. crispatus* (*n* = 9), *L. rhamnosus* (*n* = 6), *L. plantarum* (*n* = 6), *L. reuteri* (*n* = 3), *L. jensenii* (*n* = 2), *L. fermentans* (*n* = 2), *L. gasseri* (*n* = 1), and *L. bulgaricus* (*n* = 1). These results indicate that *L. crispatus* and *L. rhamnosus* are the predominant *Lactobacillus* species in vaginal samples obtained from healthy women in this study. The raw data are presented in the supplementary materials.

### 3.4. Biofilm Formation in Co-Cultures of Gardnerella and Lactobacillus

**Figure 3:**
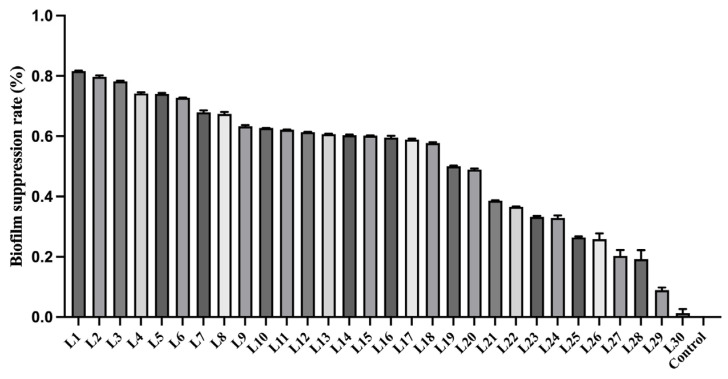
The biofilm inhibition rate after co-culture of *Lactobacillus* and Gardnerella. Description of the *Lactobacillus* species: *L. rhamnosus*: L1, L3, L10, L16, L27, and L28; *L. crispatus*: L2, L8, L13, L21, L23, L24, L25, L26, and L29; *L. reuteri*: L4, L9, and L14; *L. plantarum*: L5, L6, L7, L15, L17, and L18; *L. jensenii:* L11 and L22; *L. gasseri*: L12; *L. fermentans*: L19 and L30; *L. bulgaricus*: L20. The images are arranged in descending order from high to low, with strains numbered corresponding to this ranking. The biofilm suppression rate was calculated as follows: (1 − OD_595_, treatment/OD_595_, control) × 100%. The biofilm formed by *Gardnerella* G1 alone was used as the control, with a baseline inhibition rate of 0%. Statistical significance was determined by one-way ANOVA followed by Tukey’s post-hoc test (*p* < 0.05).

To investigate the inhibitory effect of *Lactobacillus* on *Gardnerella* biofilm, strain G1 with the strongest biofilm formation ability was selected to be cultured separately with 30 strains of *Lactobacillus* to determine the quantification of biofilm formation. Then calculate the percentage of inhibition of *Gardnerella* by each strain of *Lactobacillus* (Figure 3). Biofilms formed solely by the G1 strain served as the control group; therefore, their inhibition rate was defined as 0%. Based on the inhibitory activity of *Lactobacillus* strains against *Gardnerella* biofilms, the strains were ranked in descending order of their inhibitory effect and sequentially numbered. Statistical analysis demonstrated that different *Lactobacillus* strains exhibit distinct abilities to inhibit *Gardnerella* biofilms, with the inhibitory effect being strain-specific (*p* < 0.05).

In addition, *L. rhamnosus* (L1) and *L. crispatus* (L2) showed the strongest biofilm suppression, whereas *L. rhamnosus* (L28) and *L. crispatus* (L29) exerted the weakest inhibitory activity. Given that *L. rhamnosus* and *L. crispatus* were the most widely distributed strains in this study population, subsequent studies focused on these two species. Each species category contained one strain with strong biofilm inhibition ability and one strain with weak biofilm inhibition ability.

### 3.5. The Bacterial Activity in the Biofilm of Gardnerella and Lactobacillus

**Figure 4:**
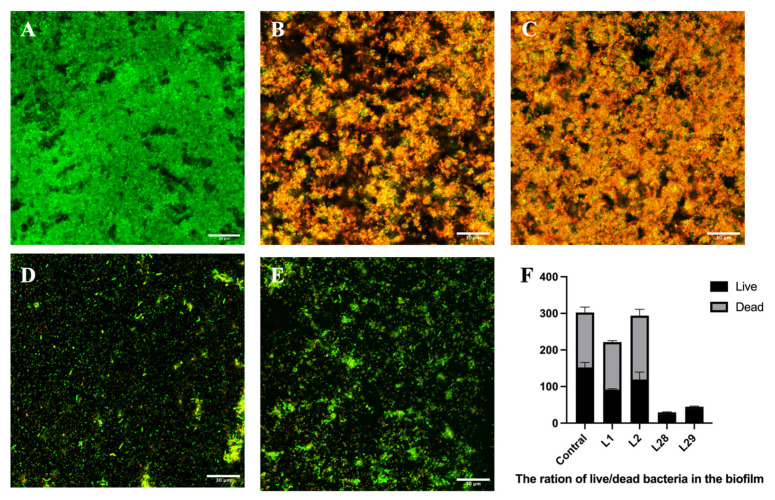
Laser confocal microscopy observation of the bacterial viability of biofilm. (**A**) Control: *Gardnerella* strain (G1) biofilm. (**B**) The biofilm formed by the co-culture of L1 and G1 strains. (**C**) The biofilm formed by the co-culture of L2 and G1 strains. (**D**) The biofilm formed by the co-culture of L28 and G1 strains. (**E**) The biofilm formed by the co-culture of L29 and G1 strains. (**A**–**E**): The rations of live/all bacteria were around 50%, 40%, 40%, 80% and 60%, respectively. (**F**) The ration of live/dead bacteria in biofilm. Green: live bacteria, Red: dead bacteria.

We selected four *Lactobacillus* strains, namely *L. rhamnosus* (L1) and *L. crispatus* (L2) (with strong biofilm-inhibiting activity) as well as *L. rhamnosus* (L28) and *L. crispatus* (L29) (with weak biofilm-inhibiting activity). Live/dead fluorescence staining was employed to observe the biofilm of G1 (the *Gardnerella* strain with the strongest biofilm-forming capacity) following interference with these *Lactobacillus* strains of varying inhibitory potency (Figure 4).

The result revealed that the control biofilm of G1 strain displayed dense, uniform green fluorescence indicative of high bacterial viability (Figure 4A), whereas L1 and L2 strain showed prominent orange fluorescence reflecting extensive bacterial death (Figure 4B,C); L28 and L29 strain exhibited sparse, scattered green/yellow puncta against a dark background (Figure 4D,E), demonstrating marked reductions in both biofilm biomass and viability. It was further supported by quantitative analysis showing a dramatic decline in live bacterial counts for all treatment groups (L1, L2, L28, L29), and a corresponding increase in dead bacteria in the L1 and L2 groups, confirming the suppression of bacterial viability by the treatments (Figure 4F).

Quantification of bacterial viability revealed that the ratios of live to total bacteria across Groups A–E were roughly 50%, 40%, 40%, 80%, and 60%, respectively. Consistent with their strong biofilm-inhibitory efficacy, *Lactobacillus* strains L1 and L2 significantly diminished the proportion of viable cells in the biofilm. Conversely, biofilms exposed to L28 and L29 intervention displayed a prominent elevation in viable bacterial proportions, alongside an extremely low incidence of dead cells. A plausible explanation for this phenomenon lies in the relatively weak inhibitory activity of L28 and L29, which fails to induce substantial *Gardnerella* cell death. Notably, it is critical to recognize that the viable bacterial population enumerated herein encompasses both *Lactobacillus* and *Gardnerella* cells, which should be taken into account when interpreting the functional implications of these viability profiles. We acknowledge the limitation of not using species-specific fluorescent tags.

### 3.6. Analysis of Biofilm Compositions

**Figure 5:**
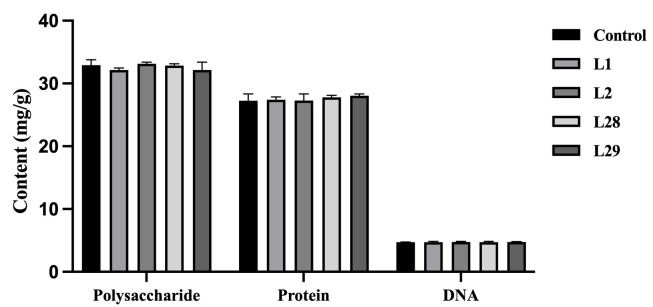
Analysis of polysaccharide, protein, and DNA levels in biofilm. Control: *Gardnerella* strain (G1). L1: Co-culture of L1 and G1 strains to form biofilm; L2: Co-culture of L2 and G1 strains to form biofilm; L28: Co-culture of L28 and G1 strains to form biofilm; L29: Co-culture of L29 and G1 strains to form biofilm. The two-way ANOVA analysis confirmed that there were no significant differences in the levels of any of these biofilm components between the groups (*p* > 0.05).

We further investigated the mechanism underlying the *Lactobacillus*-mediated inhibition of *Gardnerella* biofilms by analyzing changes in the polysaccharide, protein, and DNA content of G1 biofilms (the *Gardnerella* strain with the strongest biofilm-forming capacity) following co-culture with the four selected *Lactobacillus* strains (i.e., L1, L2, L28, and L29). Statistical analysis revealed no significant alterations in the levels of polysaccharide, protein, or DNA within the biofilms across all treatment groups (Figure 5, *p* > 0.05). Collectively, these observations demonstrate that *Lactobacillus* antagonizes *Gardnerella* biofilms through a mechanism that does not disrupt the compositional integrity of the biofilm.

### 3.7. Analysis of Supernatant Lactobacillus Compositions

**Figure 6:**
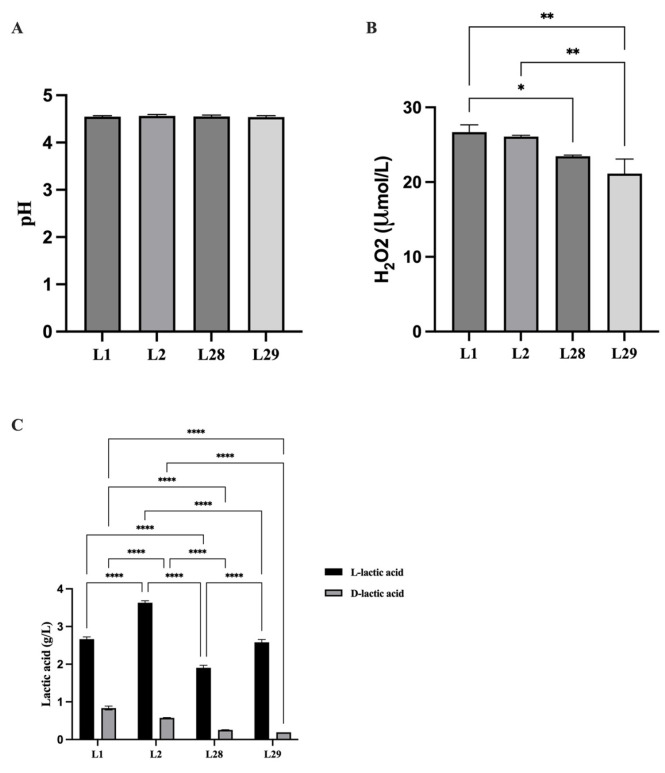
Determination of metabolic components in *Lactobacillus* supernatant. (**A**) pH. (**B**) H_2_O_2_. (**C**) Lactic acid. The one-way ANOVA analysis showed that there was no statistically significant difference in the pH values of the supernatants of different *Lactobacillus*. The statistical significance of H_2_O_2_ concentration is indicated by asterisks: L1 vs. L29 ** (*p* < 0.01), L1 vs. L28 * (*p* < 0.05), and L2 vs. L29 ** (*p* < 0.01). The two-way ANOVA analysis indicated that statistical significance (*p* < 0.05) is observed between nearly all pairwise comparisons, exception for L1 vs. L29 in the L-lactic acid concentration and L28 vs. L29 in the D-lactic acid concentration. **** *p* < 0.0001.

Subsequently, we compared the pH values, H_2_O_2_ levels, and L-/D-lactic acid concentrations in the supernatants of the four selected *Lactobacillus* strains (i.e., L1, L2, L28, and L29). The pH values of the supernatants were nearly identical across all four strains, indicating that pH is unlikely to account for the observed differences in their *Gardnerella* biofilm-inhibiting capacities (Figure 6A, *p* > 0.05). In contrast, significant inter-strain variations were detected in the H_2_O_2_ content of the supernatants (Figure 6B, *p* < 0.05), with H_2_O_2_ levels showing a positive correlation with biofilm inhibition efficacy. We further quantified L-lactic acid and D-lactic acid in the supernatants. Statistical analysis indicated that L-lactic acid levels were significantly higher than D-lactic acid levels across all strains tested, with this difference reaching statistical significance (Figure 6C, *p* < 0.05). Notably, only D-lactic acid content was consistent with the biofilm-inhibiting ability of the *Lactobacillus* strains. Despite the high levels of L-lactic acid in the supernatants, this isomer may contribute minimally to *Gardnerella* biofilm suppression. Our findings indicate that H_2_O_2_ and D-lactic acid are key bioactive components mediating the inhibitory effect of these *Lactobacillus* species on *Gardnerella* biofilm formation, while other factors such as bacteriocin may also contribute to this process and warrant further investigation. The raw data are included in the supplementary materials.

### 3.8. Time-Dependent Effects of Lactobacillus Supernatants on Gardnerella Biofilm

**Figure 7:**
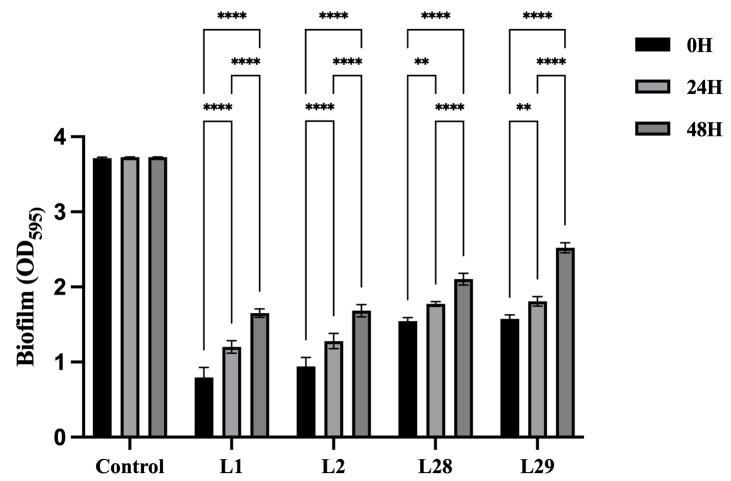
Time-dependent inhibitory effects of *Lactobacillus* supernatant on *Gardnerella* biofilms. Control: *Gardnerella* strain (G1). Treatments were defined as follows: 0 h: *Lactobacillus* supernatant and G1 were co-administered at the initiation of culture, followed by 48 h of co-incubation; 24 h: *Lactobacillus* supernatant was added after 24 h of G1 biofilm formation, with incubation continued for an additional 48 h; 48 h: *Lactobacillus* supernatant was added after 48 h of G1 biofilm formation, and incubation was prolonged for another 48 h. Excluding the control group, two-way ANOVA revealed significant intra-group temporal differences across all time points, with statistically significant increases observed between 0 h and 24 h, as well as between 24 h and 48 h (** *p* < 0.01 and **** *p* < 0.0001).

To assess the temporal impact of *Lactobacillus* supernatants on *Gardnerella* biofilm development, we quantified the biomass of G1 biofilms, by adding the supernatant of *Lactobacillus* (i.e., L1, L2, L28, L29) to the biofilm at different growth stages. As depicted in Figure 7, biofilm biomass increased in a time-dependent manner across all treatment groups, with highly significant intra-group differences observed between each time point (*p* < 0.05 for 0 h versus 24 h and 24 h versus 48 h comparisons). The inhibitory effect of the *Lactobacillus* supernatant diminished progressively as the addition time was extended. Furthermore, suppression was most pronounced when *Lactobacillus* supernatant was administered concurrently with the initial stages of biofilm formation. In addition, without the intervention of *Lactobacillus* supernatant, *Gardnerella* biofilm persisted stably without depletion.

## 4. Discussion

Our findings suggest that the production of H_2_O_2_ and D-lactic acid by specific *Lactobacillus* strains, such as *L. crispatus* L1 and *L. rhamnosus* L2, may be important contributors to the inhibition of *Gardnerella* biofilm formation observed in this study. However, given the limited number of strains tested, other metabolites and mechanisms likely play a role and warrant further investigation.

Vaginal glycogen serves as a key energy source for *Lactobacillus*, which metabolizes it to produce lactic acid; this metabolite reduces vaginal pH, thereby suppressing the growth of non-beneficial microbial flora [43]. Vaginal lactic acid exists primarily as L- and D-isomers, both of which are predominantly produced by *Lactobacillus* species [10]. Previous studies have demonstrated species-specific differences in lactic acid isomer production. Specifically, *L. crispatus* and *L. gasseri* secrete D- and L-lactic acid, while *L. iners* synthesizes only the L-isomer of lactic acid, whereas *L. jensenii* exclusively produces the D-isomer [11]. Notably, the protective capacity of the vaginal ecosystem is governed by the dominant *Lactobacillus* species within the microflora [6,44]. For instance, vaginal microbiota dominated by *L. iners* is often associated with microbial dysbiosis and reduced community stability, whereas *L. crispatus*-dominant vaginal communities are linked to enhanced health and high stability [4]. This discrepancy has been attributed to the superior protective capacity of D-lactic acid compared with L-lactic acid [11]. Correspondingly, genomic analyses targeting the four primary vaginal *Lactobacillus* species have identified differences in their D- and L-lactic acid-producing potential, with these variations correlating with the presence or absence of D- and L-lactate dehydrogenase-encoding genes. Specifically, the genome of *L. iners* completely lacks the gene encoding D-lactate dehydrogenase [11,45,46]. Although its concentration is relatively lower than that of L-lactic acid, D-lactic acid exerts a pivotal role in the maintenance of vaginal homeostasis [47].

H_2_O_2_ production by vaginal *Lactobacillus* is a key feature supporting vaginal health in individuals of reproductive age [48]. Our findings are consistent with numerous epidemiological studies demonstrating that *Lactobacillus* exerts in vivo antimicrobial effects through H_2_O_2_ synthesis [49,50,51,52]. However, H_2_O_2_ production requires molecular oxygen, an element rarely present in the vaginal microenvironment, highlighting the complexity of H_2_O_2_-mediated protection within the physiological vaginal ecosystem [53]. Notably, *Lactobacillus* strains that do not produce H_2_O_2_ also fail to generate high levels of lactic acid [46,54]. This correlation is supported by strain-specific data. For example, Hutt et al. demonstrated that H_2_O_2_ is produced by 95% of vaginal *L. crispatus* isolates, 94% of *L. jensenii* isolates, and 70% of *L. gasseri* isolates, in contrast to merely 9% of *L. iners* isolates [55]. Similarly, the primary producers of D-lactic acid in the vaginal microenvironment are *L. crispatus*, *L. jensenii*, and *L. gasseri*, a trait not shared with *L. iners* [11,45]. These findings underscore a collaborative relationship between H_2_O_2_ and lactic acid production in protective vaginal *Lactobacillus*. Atassi and Servin showed that H_2_O_2_ exhibits stronger bactericidal activity against BV-associated pathogens when combined with lactic acid [56]. Strus et al. demonstrated that culture supernatants derived from H_2_O_2_-producing *Lactobacillus* strains exert the strongest inhibitory effects against both bacteria and yeasts, followed by supernatants from non-H_2_O_2_-producing strains and pure H_2_O_2_, indicating that *Lactobacillus* antimicrobial activity arises from the integration of multiple mechanisms [57], with H_2_O_2_ playing an important but non-essential role [58]. Beyond direct antimicrobial action, H_2_O_2_ interacts with vaginal microorganisms and the vaginal mucosa, including epithelial and immune cells [59]. It stimulates *Lactobacillus* growth and their antagonistic activity against pathogens, while enhancing the antimicrobial efficacy of host protective factors such as muramidase, lactoferrin, and metabolites secreted by epithelial cells [60]. Additionally, H_2_O_2_ disrupts the balance of pro-oxidants and antioxidants in pathogen cells, creating oxidative stress that potentiates antibiotic efficacy [61]. These data indicate that the mechanism by which H_2_O_2_ exerts its antibacterial effect is complex and warrants further investigation. Collectively, these observations indicate that H_2_O_2_ exerts its antibacterial effects through a complex interplay of direct microbial inhibition, synergy with other *Lactobacillus* metabolites, modulation of host defenses, and enhancement of antibiotic activity. Further research is required to fully elucidate the multifaceted mechanisms by which H_2_O_2_ contributes to vaginal health. In fact, apart from H_2_O_2_ and D-lactic acid, there are other metabolites in the supernatant of *Lactobacillus* that play a systemic role in inhibiting the biofilm of Gardnerella. Future research should also focus on other metabolites.

Bacterial biofilms are clusters of microorganisms that adhere to the vaginal epithelium, encased within a self-synthesized extracellular matrix composed of proteins, polysaccharides, and extracellular DNA [62,63]. Owing to the hypoxic microenvironment and nutrient limitation within biofilms, the bacteria grow slowly and enter a dormant state; these factors further alter the molecular targets of antibiotic action [64]. Therefore, when the *Gardnerella* biofilm is antagonized by *Lactobacillus*, the composition of the biofilm will not change. *Lactobacillus* supernatant exhibited the strongest inhibitory effect when it was added at the initiation of *Gardnerella* biofilm culture. This result is consistent with our previous research findings [65]. We hypothesize that *Lactobacillus* can also inhibit the formation of biofilms by replacing the adhesion sites of *Gardnerella* [66,67]. Moreover, the adhesion and colonization of *Gardnerella* is precisely the initial step in the biofilm formation [6,68]. According to findings from Zhang et al., the supplementation of lactic acid and H_2_O_2_ into *Gardnerella* culture media resulted in downregulation of multiple genes, with the majority of these genes being associated with biofilm biosynthesis and epithelial cell adherence [69]. While the initial inoculum volume was adjusted according to the culture scale, the consistent inhibitory trends observed across different assays support the robust antibiofilm potential of *Lactobacillus*.

## 5. Conclusions

A vaginal microbiome dominated by *Lactobacillus* species is crucial for resisting *Gardnerella*-associated dysbiosis. In the present study, the antagonistic mechanism of *Lactobacillus* against *Gardnerella* biofilms was investigated, providing novel insights for probiotic application. *L. crispatus* and *L. rhamnosus* may suppress *Gardnerella* biofilm formation mainly through their metabolic products H_2_O_2_ and D-lactic acid. Notably, this inhibitory effect is independent of changes in the components of the *Gardnerella* biofilm. The present study broadens our understanding of the antagonistic functions of *Lactobacillus* within the vaginal microbiome, offering new experimental evidence for the metabolite-driven protective function of probiotic *Lactobacillus*. Furthermore, it provides a solid basis for screening high-efficacy probiotic strains and developing targeted therapeutic strategies for BV.

### Limitation

Some limitations of this study should be acknowledged. First, the current methods for measuring biofilms are unable to distinguish *Lactobacillus* from *Gardnerella* cells. Furthermore, the live/dead staining method employed lacks species-specific discriminatory capacity. Second, the analyses were restricted to in vitro biofilms grown on abiotic surfaces, which may not fully reflect the in vivo physiological context. Third, only one *Gardnerella* strain was evaluated in this study, limiting the generalizability of the findings. Additionally, the quantification of biofilm components was normalized by wet weight rather than dry weight. While dry weight is generally a more accurate metric for comparing strains with varying water content, obtaining consistent dry mass from 6-well plates was technically challenging due to the limited biomass recovered. Finally, the limited inhibitory effect of the therapy on pre-formed *Gardnerella* biofilms may constrain its practical clinical application.

## Data Availability

The original contributions presented in this study are included in the article/supplementary material. Further inquiries can be directed to the corresponding author.

## Supplementary Materials

The following supporting information can be downloaded at: https://www.mdpi.com/article/10.3390/microorganisms14030569/s1. Supplementary table presents the quantification of Gardnerella biofilm, quantification of Lactobacillus–Gardnerella co-culture biofilm, and compositional analysis of Lactobacillus supernatant. Raw data are presented in this table.
